# In Vitro Maturation of Bone Marrow-Derived Dendritic Cells via STING Activation for T Cell Priming

**DOI:** 10.3390/cancers17213497

**Published:** 2025-10-30

**Authors:** Busra Buyuk, Kaiming Ye

**Affiliations:** 1 Department of Biomedical Engineering, Center of Biomanufacturing for Regenerative Medicine, Watson College of Engineering and Applied Science, State University of New York (SUNY), Binghamton, NY 13902, USA; 2 Department of Bioengineering, Recep Tayyip Erdogan University, Rize 53100, Türkiye

**Keywords:** bone marrow-derived dendritic cells, STING agonists, granulocyte–macrophage colony-stimulating factor, interleukin-4, antigen presentation, CD8-positive T-cell proliferation, naïve T cells, cyclic dinucleotides

## Abstract

**Simple Summary:**

Dendritic cells (DCs) are crucial for activating the immune system and have great potential in cancer immunotherapy. This study aimed to generate DCs from mouse bone marrow, activate them through the STING signaling pathway, and evaluate their ability to stimulate T cells. Optimized culture conditions and a STING agonist promoted the formation of mature and functional DCs capable of triggering strong CD8^+^ T cell responses. These findings highlight the potential of STING-activated DCs to enhance anti-tumor immunity and pave the way for developing personalized and effective cancer treatment strategies.

**Abstract:**

Objective: Dendritic cells (DCs) are the most potent antigen-presenting cells, serving as a bridge between innate and adaptive immunity. Activation of the stimulator of interferon genes (STING) pathway by pathogen-derived DNA induces type I interferon responses and promotes CD8^+^ cytotoxic T cell activity. This study aimed to establish a protocol for generating immature DCs from murine bone marrow, optimize their maturation in vitro with a STING agonist, and evaluate their ability to prime naïve T cells for potential use in cancer immunotherapy. Methods: Bone marrow cells from C57BL/6 mice were differentiated into immature DCs under growth factor–supplemented conditions. Maturation was induced using a STING agonist and B16 tumor-derived DNA. Naïve CD4^+^ and CD8^+^ T cells were isolated via magnetic-activated cell sorting (MACS) and co-cultured with the stimulated DCs. Culture conditions were optimized to enhance DC maturation efficiency, and T cell proliferation was assessed following co-culture. Results: Optimization of the culture system markedly increased the yield of mature DCs. Importantly, co-culture of STING agonist-stimulated DCs with naïve T cells resulted in strong CD8^+^ T cell proliferation, indicating effective priming. Conclusions: These findings demonstrate the feasibility of generating functional DCs in vitro and highlight their capacity to prime T cells through STING pathway activation. This proof-of-concept supports the development of DC-based platforms as a promising strategy for novel cancer immunotherapies.

## 1. Introduction

Dendritic cells (DCs) play a critical role in the innate immune system [1,2,3,4]. They are the most potent antigen-presenting cells, functioning as a vital bridge between the innate and adaptive immune systems [5,6]. Their capacity to capture, process, and present antigens to T cells is fundamental for initiating immune responses that include the activation of CD8^+^ T cells, which are essential for combating viral infections and cancer [7]. The differentiation of DCs from hematopoietic stem cells in the bone marrow can be induced in vitro using cytokines such as granulocyte–macrophage colony-stimulating factor (GM-CSF) and interleukin-4 (IL-4) [8,9,10]. These cytokines play a key role in promoting the maturation of immature DCs by enhancing their ability to present antigens and activate T cells [11].

The differentiation and maturation of DCs are regulated by several factors, including the expression of surface markers such as CD11c, CD80, and MHC class II [12,13,14,15]. These, along with other markers, serve as indicators of the antigen-presenting capacity of DCs. GM-CSF and IL-4 are widely employed to induce DC differentiation; however, the optimal cytokine concentrations required to achieve the highest level of antigen-presenting function remain unclear [12,16]. A deeper understanding of how these cytokines influence DC maturation could significantly improve their application in immunotherapy, particularly in cancer treatment, by enhancing their ability to effectively activate immune cells.

The stimulator of interferon genes (STING) pathway is also a key regulator of immune responses [17,18]. STING is a cytosolic receptor that detects pathogenic DNA within the cell and triggers a signaling cascade that leads to the production of type I interferons [17,19]. These interferons activate immune cells, including DCs, and foster an inflammatory environment that amplifies immune responses [20]. Activation of the STING pathway enhances the antigen-presenting functions of DCs and promotes CD8^+^ T cell activation, which plays an essential role in defending against pathogens and tumors [21,22].

Although the effects of STING activation on DC function are well established, the optimal conditions for combining STING activation with cytokine-driven DC differentiation remain largely unexplored. Therefore, this study aims to investigate the effects of varying GM-CSF and IL-4 concentrations on DC differentiation, focusing on the expression of key surface markers (CD11c, CD80, and MHC II). Furthermore, it evaluates how activation of the STING pathway influences DC maturation and their capacity to stimulate T cells. In this study, bone marrow–derived DCs were generated using GM-CSF and IL-4 and subsequently exposed to different concentrations of a STING agonist and tumor-derived DNA. We assessed the expression of CD11c, CD80, and MHC II, as well as the ability of mature DCs (stimulated with STING agonist and tumor DNA) to induce CD8^+^ T cell activation and proliferation in co-culture experiments. This research aims to train naïve T cells in vitro by defining the most effective conditions for DC maturation and STING activation, contributing to the advancement of personalized and efficient immunotherapy strategies.

## 2. Materials and Methods

### 2.1. Isolation and Differentiation of Bone Marrow-Derived Dendritic Cells (BMDCs)

#### 2.1.1. Bone Marrow Extraction

All animal experiments were conducted at Binghamton University under an IACUC-approved protocol in compliance with American Veterinary Medical Association guidelines. Female C57BL/6 mice (6–8 weeks, The Jackson Laboratory, Bar Harbor, ME, USA) were euthanized by CO_2_ inhalation. Femurs, tibias, and humeri were harvested under sterile conditions and transferred to cold isolation buffer (DPBS supplemented with 2% FBS and 2 mM EDTA). Bone marrow cells were flushed using a 27-gauge needle with RPMI-1640 medium, passed through a 70 µm strainer, and centrifuged at 1500 rpm for 8 min. Red blood cells were lysed using RBC lysis buffer (150 mM NH_4_Cl, 10 mM KHCO_3_, 0.1 mM EDTA, pH 7.2–7.4). Viable cells were counted by trypan blue exclusion.

#### 2.1.2. DC Culture

Cells were plated at a density of 1 × 10^6^ cells/mL in 100 mm tissue culture-treated dishes containing 10 mL of DC culture medium [RPMI-1640 supplemented with 10% heat-inactivated FBS, 2 mM L-glutamine, 50 µM β-mercaptoethanol, 1× penicillin/streptomycin, 20 ng/mL GM-CSF, and 20 ng/mL IL-4] and incubated at 37 °C in a humidified 5% CO_2_ atmosphere. On day 3, cultures were visually examined under an inverted phase-contrast microscope. Floating and loosely adherent cells, enriched for dendritic cells, were gently collected, while adherent cells representing macrophage-like populations were left behind. Fresh medium containing GM-CSF and/or IL-4 was added to the remaining cells to maintain differentiation. On day 6, the same procedure was repeated to collect non-adherent immature DCs for subsequent experiments.

#### 2.1.3. Cytokine Optimization

To determine optimal cytokine conditions, four regimens were tested over 10 days:

1. GM-CSF (20 ng/mL, days 0, 3, 6, 8).
2. GM-CSF (20 ng/mL, days 0, 3) followed by GM-CSF (10 ng/mL) + IL-4 (10 ng/mL, days 6, 8).
3. GM-CSF (20 ng/mL, days 0, 3) followed by GM-CSF (10 ng/mL, days 6, 8).
4. Step-down GM-CSF: 20 → 10 → 5 → 2.5 ng/mL on days 0, 3, 6, and 8.

Cells were harvested on day 10 for flow cytometric analysis.

### 2.2. Immunostaining and Flow Cytometry

Cells (1 × 10^6^) were incubated with Fc block (anti-CD16/CD32, BioLegend (San Diego, CA, USA) for 15 min on ice, followed by staining with fluorophore-conjugated antibodies: CD11c, CD80, MHC II, CD4, CD8, CD25, and CD69 BioLegend (San Diego, CA, USA) CD11c and CD80 were diluted 1:100, and MHC II at 1:300. After staining for 30 min on ice, cells were washed, fixed with 4% paraformaldehyde, and analyzed using a BD flow cytometer at the Analytical and Diagnostics Laboratory, Binghamton University. Flow cytometry data were analyzed using FlowJo software (BD Biosciences, San Jose, CA, USA).

Antibodies used:

- CD11c (APC, 1:100, BioLegend (San Diego, CA, USA)
- CD80 (Alexa Fluor 647, 1:100, BioLegend (San Diego, CA, USA)
- MHC II (PE, 1:300, BioLegend (San Diego, CA, USA)
- CD4 (APC, 1:100, BioLegend (San Diego, CA, USA)
- CD8 (APC, 1:100, BioLegend (San Diego, CA, USA)
- CD25 (PE, 1:100, BioLegend (San Diego, CA, USA)
- CD69 (PE, 1:100, BioLegend (San Diego, CA, USA)

### 2.3. STING Pathway Activation and Stimulation Design

BMDCs cultured under optimized cytokine conditions were stimulated on day 9 with the synthetic STING agonist 2′3′-c-di-AM(PS)_2_ (Rp,Rp) VacciGrade™ InvivoGen (San Diego, CA, USA) at concentrations of 20 ng/mL, 2.5 µg/mL, and 5 µg/mL for 24 h. These concentrations were selected based on previously reported dose–response characteristics of cyclic dinucleotides (CDNs) in murine dendritic cells, covering both low (nanogram) and high (microgram) activation ranges of the STING pathway. Control cells received no agonist. On day 10, CD80 and MHC II expression were analyzed by flow cytometry to determine dose-dependent effects.

Based on these results, 5 µg/mL was selected as the optimal concentration for subsequent functional assays. To compare the ability of the synthetic agonist and tumor-derived DNA to stimulate T cells, BMDCs were treated with either 5 µg/mL STING agonist or 5 µg/mL B16 melanoma DNA (delivered with a lipid-based transfection reagent). B16 DNA was not included in the dose–response assay for DC maturation; instead, it was used exclusively in T cell co-culture experiments as a functional comparator to the synthetic agonist.

### 2.4. Spleen Harvest, Digestion, and T Cell Isolation

Spleens were collected from C57BL/6 mice under sterile conditions, minced, and digested with collagenase IV (1 mg/mL) and DNase I (100 µg/mL) at 37 °C for 30 min. Suspensions were filtered through 70 µm strainers, red blood cells lysed, and cells washed. Splenocytes were resuspended in FACS buffer (DPBS, 2% FBS, 25 mM HEPES, 2 mM EDTA).

Naïve CD4^+^ and CD8^+^ T cells were isolated using the MojoSort™ magnetic-activated cell sorting (MACS) system BioLegend (San Diego, CA, USA). Briefly, splenocytes (1 × 10^7^ cells) were incubated with biotinylated anti-CD4 or anti-CD8 antibody cocktails for 15 min on ice, followed by streptavidin nanobeads for an additional 15 min. Magnetically labeled cells were separated according to the manufacturer’s instructions. The isolated naïve T cells were resuspended in complete RPMI medium and used for subsequent co-culture assays.

### 2.5. DC–T Cell Co-Culture

BMDCs stimulated with 5 µg/mL STING agonist were co-cultured with naïve CD4^+^ or CD8^+^ T cells at a DC:T ratio of 1:5 for 3 days in complete RPMI medium.

### 2.6. CFSE Proliferation Assay

Naïve T cells (CD4 and CD8) were labeled with CFSE (0.5 µM, BioLegend (San Diego, CA, USA) for 20 min at room temperature in the dark. Labeling was quenched with FBS, and cells were washed. After co-culture with BMDCs, CFSE dilution was measured by flow cytometry to evaluate proliferation.

### 2.7. T Cell Co-Culture and Proliferation Assay

BMDCs stimulated with 5 µg/mL STING agonist or 5 µg/mL B16 DNA were co-cultured with naïve CD4^+^ or CD8^+^ T cells at a 1:5 (DC:T) ratio for 3 days. Prior to co-culture, T cells were labeled with CFSE to monitor proliferation. CD25 was used as an activation marker for CD4^+^ T cells, and CD69 was used for CD8^+^ T cells. Flow cytometry was performed to assess activation and proliferation.

## 3. Results

### 3.1. Optimization of Cytokine Conditions for BMDC Differentiation

**Figure 1:**
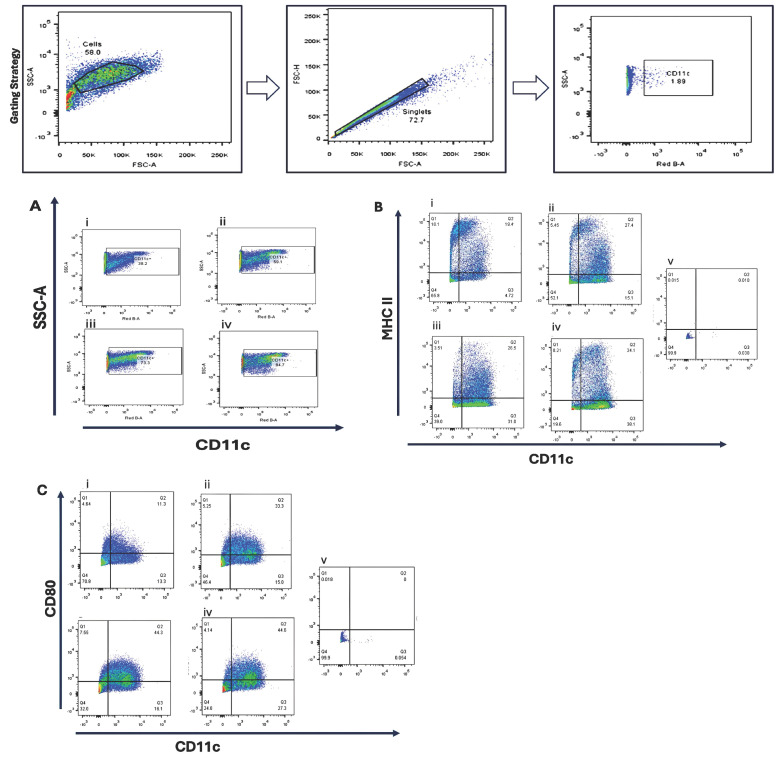
Phenotypic characterization of bone marrow-derived dendritic cells (BMDCs) cultured under different cytokine conditions. Bone marrow cells were cultured for 10 days in four different cytokine conditions to evaluate dendritic cell (DC) differentiation and maturation: (**i**) Condition 1—Stepwise reduction of GM-CSF, starting at 20 ng/mL on day 0, then decreasing to 10 ng/mL (day 3), 5 ng/mL (day 6), and 2.5 ng/mL (day 8); (**ii**) Condition 2—20 ng/mL GM-CSF (day 0, 3); 10 ng/mL GM-CSF (day 6, 8); (**iii**) Condition 3—20 ng/mL GM-CSF; (day 0, 3, 6, 8); (**iv**) Condition 4—20 ng/mL GM-CSF (day 0, 3), followed by 10 ng/mL GM-CSF + 10 ng/mL IL-4 (day 6, 8). Flow cytometry was performed on day 10 to assess the expression of dendritic cell surface markers. (**A**) CD11c expression as a general DC marker; (**B**) MHC class II expression, indicating antigen-presenting capability; (**C**) CD80 expression, representing DC maturation status. Unstained bone marrow cells (**v**) were used as negative controls for gating. Representative flow cytometry plots are shown.

Bone marrow-derived cells were cultured under four cytokine supplementation regimens to optimize dendritic cell differentiation. Flow cytometric analysis revealed that GM-CSF (10 ng/mL) combined with IL-4 (10 ng/mL) produced the highest proportion of CD11c^+^ cells (84.7%) and increased MHC II expression (34.1%) compared with GM-CSF alone (26–27%). CD80 expression was enhanced by higher GM-CSF concentrations, suggesting a dose-dependent effect on dendritic cell maturation Figure 1.

### 3.2. STING Agonist-Induced Maturation of Dendritic Cells

**Figure 2:**
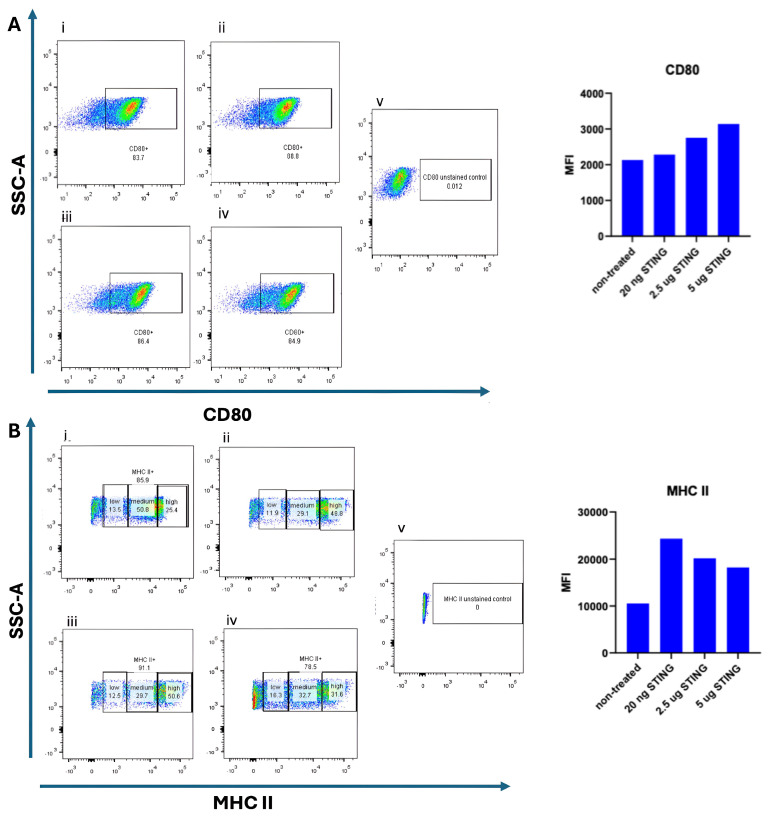
Dose-dependent maturation of BMDCs induced by STING agonist. Bone-marrow-derived dendritic cells (BMDCs) were cultured for 9 days in the presence of 10 ng/mL GM-CSF and 10 ng/mL IL-4. On day 9, the cells were stimulated for 24 h with increasing concentrations of the STING agonist 2′3′-c-di-AM(PS)_2_ (BraRR VacciGrade™, InvivoGen, San Diego, CA, USA). Flow cytometry was performed to evaluate the expression of CD80 and MHC II and their corresponding median fluorescence intensity (MFI) values. In panel (**A**), CD80 expression is shown under five conditions:(**i**) unstimulated/non-treated cells; (**ii**) cells treated with 20 ng/mL STING agonist; (**iii**) cells treated with 2.5 µg/mL STING agonist; (**iv**) cells treated with 5 µg/mL STING agonist; and (**v**) unstained control cells. In panel (**B**), MHC II expression is shown under the same conditions: (**i**) unstimulated/non-treated cells; (**ii**) 20 ng/mL STING agonist; (**iii**) 2.5 µg/mL STING agonist; (**iv**) 5 µg/mL STING agonist; and (**v**) unstained control cells. Representative flow cytometry plots and corresponding MFI bar graphs are presented for each condition.

To assess whether STING pathway activation promotes dendritic cell (DC) maturation, bone marrow-derived DCs (BMDCs) were cultured for 9 days in the presence of 10 ng/mL GM-CSF and 10 ng/mL IL-4. On day 9, cells were stimulated with the STING agonist 2′3′-c-di-AM(PS)_2_ (Rp,Rp) at varying concentrations (20 ng/mL, 2.5 µg/mL, and 5 µg/mL) and incubated for an additional 24 h. The 20 ng/mL dose was included as a low-range exposure, intended to evaluate whether minimal stimulation could initiate early activation markers. The 2.5 µg/mL and 5 µg/mL concentrations represent intermediate and high doses, respectively, based on prior literature and product recommendations for in vitro activation of mouse BMDCs. Untreated BMDCs served as negative controls. Flow cytometry was performed to evaluate the surface expression of maturation markers CD80 and MHC II. As shown in Figure 2, CD80 expression was detected on 83.7% of untreated DCs. The elevated baseline CD80 expression in untreated DCs suggests a semi-mature phenotype typical of prolonged GM-CSF/IL-4 exposure; subsequent STING activation further enhanced their maturation and antigen-presenting potential. Upon stimulation with 20 ng/mL STING agonist, CD80 expression slightly increased to 88.8%. At higher doses of the STING agonist (2.5 µg/mL and 5 µg/mL), a noticeable increase in the median fluorescence intensity (MFI) of CD80 was observed Figure 2B, suggesting a dose-dependent upregulation of this maturation marker. However, the difference in CD80 MFI between the 2.5 µg/mL and 5 µg/mL doses was minimal, indicating a potential saturation effect. The modest difference in CD80 intensity at 20 ng/mL STING agonist compared to untreated DCs may reflect limited activation at low concentrations. We speculate that the continuous presence of GM-CSF and IL-4 throughout the culture period may influence the degree of CD80 upregulation upon STING stimulation. MHC II expression was also examined following STING agonist treatment. As depicted in Figure 2, untreated DCs exhibited the lowest MFI of MHC II expression. Interestingly, stimulation with the lowest dose (20 ng/mL) of STING agonist resulted in the highest MFI for MHC II, while treatment with 2.5 µg/mL yielded a slightly lower, but still elevated, MFI. The highest dose (5 µg/mL) did not further enhance MHC II intensity and showed a marginal decrease compared to 2.5 µg/mL. These findings indicate that while STING activation can upregulate MHC II expression in DCs, a higher agonist concentration does not necessarily correlate with greater activation, possibly due to receptor saturation or negative feedback mechanisms.

### 3.3. CD4^+^ and CD8^+^ T Cell Activation and Proliferation

**Figure 3:**
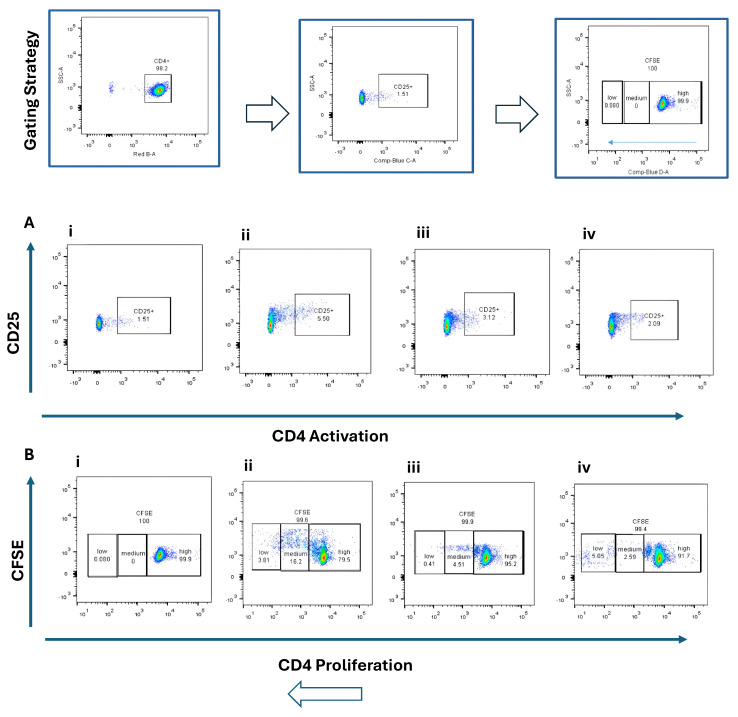
Activation and proliferation of CD4^+^ naïve T cells following co-culture with dendritic cells (DCs). Representative flow cytometry plots show (**A**) CD4^+^ T cell activation assessed by CD25 expression and (**B**) CD4^+^ T cell proliferation assessed by CFSE dilution. Four experimental conditions were analyzed: (**i**) CD4^+^ naïve T cells alone (control), (**ii**) T cells co-cultured with unstimulated DCs, (**iii**) T cells co-cultured with DCs stimulated with 5 µg/mL B16 DNA, and (**iv**) T cells co-cultured with DCs stimulated with 5 µg/mL STING agonist.

**Figure 4:**
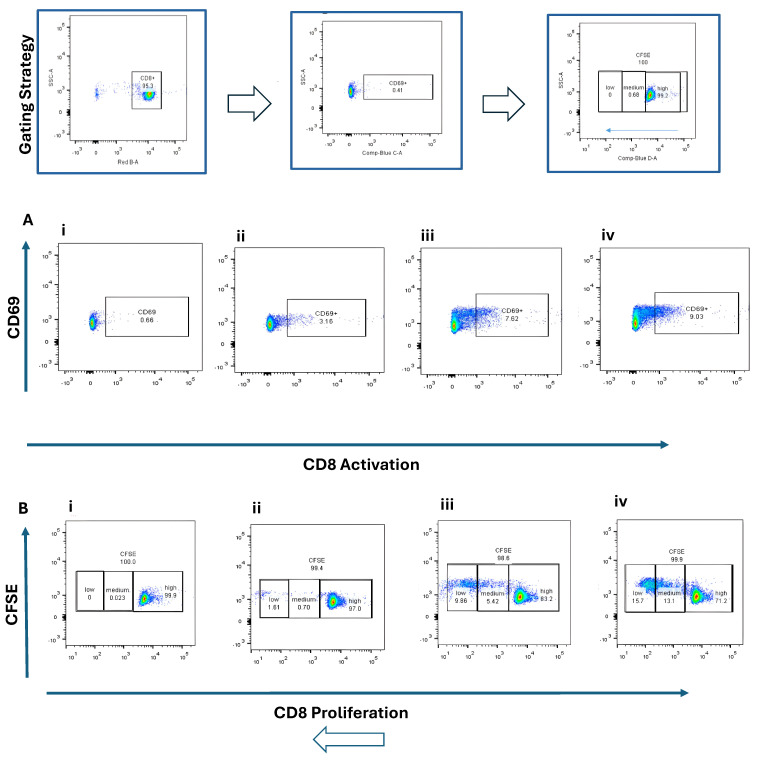
Activation and proliferation of CD8^+^ naïve T cells following co-culture with dendritic cells (DCs). Representative flow cytometry plots show (**A**) CD8^+^ T cell activation assessed by CD69 expression and (**B**) CD8^+^ T cell proliferation assessed by CFSE dilution. Four experimental conditions were analyzed: (**i**) CD8^+^ naïve T cells alone (control), (**ii**) T cells co-cultured with unstimulated DCs, (**iii**) T cells co-cultured with DCs stimulated with 5 µg/mL B16 DNA, and (**iv**) T cells co-cultured with DCs stimulated with 5 µg/mL STING agonist.

Naïve CD4^+^ and CD8^+^ T cells were co-cultured with BMDCs stimulated by STING agonist (5 µg/mL) or B16 DNA (5 µg/mL) for 3 days. CD4^+^ T cells exhibited modest activation (CD25^+^, ~2–5%) (Figure 3A) and limited proliferation (Figure 3B) compared with CD8^+^ T cells, which demonstrated robust activation (CD69^+^, ~9%) (Figure 4(Aiv)) and higher proliferation (15.7% + 13.1%) (Figure 4(Biv)) when co-cultured with STING-activated BMDCs. B16 DNA-stimulated BMDCs also induced T cell proliferation, though to a slightly lower degree than synthetic agonist stimulation (Figure 3(Biii) and Figure 4(Biii)).

## 4. Discussion

In this study, we demonstrated that bone marrow-derived dendritic cells (BMDCs) can be efficiently generated in vitro using GM-CSF and IL-4, and subsequently matured through STING pathway activation. Our findings highlight three key observations: (i) IL-4 supplementation enhances MHC II expression and supports optimal DC maturation, (ii) STING agonists promote upregulation of maturation markers CD80 and MHC II, and (iii) STING-activated BMDCs are capable of priming naïve T cells, with CD8^+^ T cells exhibiting robust activation and proliferation compared with CD4^+^ T cells.

The cytokine optimization results suggest that while GM-CSF promotes DC expansion, IL-4 synergistically enhances antigen-presenting capacity, consistent with previous studies showing IL-4’s role in upregulating MHC II and co-stimulatory molecules [23,24]. This balance is critical for generating BMDCs that are both abundant and immunologically competent.

STING activation with synthetic cyclic dinucleotides or tumor-derived DNA led to upregulation of maturation markers, reinforcing the central role of cytosolic DNA sensing in dendritic cell biology [25]. Interestingly, the low-dose agonist preferentially enhanced MHC II expression, while higher doses increased CD80, indicating that STING signaling intensity may differentially regulate costimulatory versus antigen-presenting pathways. B16 DNA stimulation yielded comparable effects, supporting the hypothesis that tumor-derived DNA fragments can act as natural STING ligands within the tumor microenvironment.

Importantly, STING-activated BMDCs effectively primed naïve T cells in vitro. CD8^+^ T cells exhibited significant clonal expansion, in line with their established role as cytotoxic effectors in antitumor immunity. In contrast, CD4^+^ T cells showed modest activation and limited proliferation, suggesting that additional signals (e.g., IL-2, CD40L-mediated DC licensing) may be required for full helper T cell expansion [26,27,28]. These findings are consistent with prior reports that STING activation enhances CD8^+^ T cell priming through type I interferon secretion and upregulation of costimulatory molecules [29,30].

From a translational perspective, our results underscore the potential of STING agonists as adjuvants in dendritic cell-based cancer vaccines [30]. By improving the immunogenicity of DCs and their capacity to prime cytotoxic T lymphocytes, STING activation may help overcome immunosuppressive barriers in the tumor microenvironment. Furthermore, the ability of tumor-derived DNA to elicit similar responses suggests that endogenous DNA fragments could contribute to spontaneous antitumor immunity [10].

In conclusion, this work establishes an in vitro platform for DC maturation and T cell priming through STING activation. Future in vivo studies are warranted to evaluate the therapeutic efficacy of STING-stimulated DC vaccines, the durability of induced T cell responses, and potential synergistic effects with checkpoint inhibitors or tumor-associated antigen delivery.

## 5. Conclusions

In this study, we established an in vitro model for the maturation of murine bone marrow-derived dendritic cells (BMDCs) via STING pathway activation and evaluated their ability to prime T cells. IL-4 supplementation enhanced MHC II expression, while STING agonist treatment further upregulated CD80 and promoted the functional maturation of DCs capable of activating naïve CD8^+^ T cells. The observed CD8^+^ T cell proliferation and activation confirmed the immunostimulatory potential of STING-activated DCs. These findings highlight the feasibility of using this in vitro platform to study DC–T cell interactions and to guide the design of dendritic cell–based immunotherapies.

Although the current work represents a proof-of-concept using limited biological replicates, the reproducible phenotypic patterns support the robustness of the findings. Future studies should focus on validating these results in vivo using tumor models such as B16 melanoma to evaluate antitumor efficacy, persistence of T cell responses, and long-term immune memory. Moreover, optimization of DC–T cell co-culture parameters (e.g., cell ratio, cytokine supplementation) and the inclusion of antigen-specific stimulation strategies may enhance the translational potential of this approach. Finally, integrating STING-activated DCs with combinatorial immunotherapies—such as immune checkpoint inhibitors, oncolytic viruses, or adoptive T cell therapies—could provide a powerful framework for next-generation cancer immunotherapy.

## Data Availability

The datasets generated and analyzed during the current study are available from the corresponding author upon reasonable request.
